# Differential effects of specific antipsychotic drugs on metabolic markers and diabetes: A register-based study on 4,909 patients with schizophrenia

**DOI:** 10.1192/j.eurpsy.2023.2270

**Published:** 2023-07-19

**Authors:** M. A. Sørensen, O. Köhler-Forsberg, C. G. Rohde, A. A. Danielsen, O. Mors

**Affiliations:** 1 Psychosis Research Unit; 2 Depression and Anxiety Research Unit, Aarhus University Hospital - Psychiatry, Aarhus C, Denmark

## Abstract

**Introduction:**

Antipsychotics (AP) are used as the primary pharmaceutical treatment for schizophrenia. Randomised clinical trials (RCT) show that the initiation of AP treatment often induces side effects such as substantial weight gain and metabolic disturbances, including an increased risk of type-2 diabetes (DM). However, the limitations of RCTs are often small cohorts that only represent a minority of patients with schizophrenia seen in everyday clinical settings leading to selection bias. Many RCTs are also limited by a short follow-up time, as evaluation of metabolic disturbances requires months to years of observation.

**Objectives:**

Within a large cohort of real-world patients with long-term follow-up, we aim to study the differential metabolic side effects of specific antipsychotic drugs.

**Methods:**

We performed a retrospective cohort study using the electronic patient record system “MidtEPJ”, which contains data from blood samples and medication usage from all patients registered with a schizophrenia diagnosis (ICD-10 code DF20) in the central region of Denmark from 2016-2022. Patients were followed from September 2016 (for patients with a schizophrenia diagnosis before this date) or their first schizophrenia diagnosis. The exposure is treatment with AP medication. Outcomes of interest are the development of DM, defined as a diagnosis of DM or usage of anti-diabetic medication, and changes in HbA1c, glucose, and cholesterol levels (high-density lipoprotein [HDL], low-density lipoprotein [LDL], total cholesterol and triglycerides). We performed cox regression analyses to study the associations between specific AP compounds with the differential risk for developing DM and changes in metabolic markers.

**Results:**

We identified 4909 individual patients with a schizophrenia diagnosis from October 1st, 2016, to September 30th, 2022. AP was subscribed to 4609 of these patients. The results will be presented at the 2023 EPA Congress.

**Conclusions:**

Our results will be discussed at the conference.

**Disclosure of Interest:**

None Declared

